# Axial de-scanning using remote focusing in the detection arm of light-sheet microscopy

**DOI:** 10.21203/rs.3.rs-3338831/v1

**Published:** 2023-10-03

**Authors:** HASSAN DIBAJI, ALI KAZEMI NASABAN SHOTORBAN, MAHSA HABIBI, RACHEL M GRATTAN, SHAYNA LUCERO, DAVID J. SCHODT, KEITH A. LIDKE, JONATHAN PETRUCCELLI, DIANE S. LIDKE, SHENG LIU, TONMOY CHAKRABORTY

**Affiliations:** 1 Department of Physics and Astronomy, University of New Mexico, Albuquerque, New Mexico 87131, USA; 2 Comprehensive Cancer Center, University of New Mexico Health Sciences Center, Albuquerque, New Mexico, 87131, USA; 3 Department of Pathology, University of New Mexico Health Science Center, Albuquerque, NM, USA; 4 Department of Physics, University at Albany–State University of NewYork,1400 Washington Avenue, Albany, NY 12222, USA

## Abstract

The ability to image at high speeds is necessary in biological imaging to capture fast-moving or transient events or to efficiently image large samples. However, due to the lack of rigidity of biological specimens, carrying out fast, high-resolution volumetric imaging without moving and agitating the sample has been a challenging problem. Pupil-matched remote focusing has been promising for high NA imaging systems with their low aberrations and wavelength independence, making it suitable for multicolor imaging. However, owing to the incoherent and unpolarized nature of the fluorescence signal, manipulating this emission light through remote focusing is challenging. Therefore, remote focusing has been primarily limited to the illumination arm, using polarized laser light for facilitating coupling in and out of the remote focusing optics. Here we introduce a novel optical design that can de-scan the axial focus movement in the detection arm of a microscope. Our method splits the fluorescence signal into S and P-polarized light and lets them pass through the remote focusing module separately and combines them with the camera. This allows us to use only one focusing element to perform aberration-free, multi-color, volumetric imaging without (a) compromising the fluorescent signal and (b) needing to perform sample/detection-objective translation. We demonstrate the capabilities of this scheme by acquiring fast dual-color 4D (3D space + time) image stacks, with an axial range of 70 μm and camera limited acquisition speed. Owing to its general nature, we believe this technique will find its application to many other microscopy techniques that currently use an adjustable Z-stage to carry out volumetric imaging such as confocal, 2-photon, and light sheet variants.

Fast 3D positioning or scanning of an optical system’s focal point or focal plane has the potential to transform many areas of BioPhotonics, especially those that require studying the complex dynamics of living organisms. Processes like investigation of neuronal activities of the brain, blood flow in the heart, and cell signaling require high-speed volumetric imaging^1–3^. However, volumetric imaging requires an axial scan either through the translation of the sample or the detection objective lens (Fig. 1a). Such axial translations result in imaging modalities that are often slow with speeds limited to a few hundred Hz^4–6^. Additionally, with fragile samples, such as an expanded sample in hydrogel^7^, fast movements of the sample stage may agitate the sample and induce distortions when collecting volumetric images. To avoid the slow translation of bulky objectives or the sample stages, several attempts, employing variable-focus (vari-focus) lenses, mechanical mirrors, and acousto-optics modulators have been proposed to refocus the light for 3D imaging. However, they all suffer from unacceptable aberrations introduced by the focusing elements. A large category of those techniques utilize different types of tunable lenses such as ferroelectric liquid crystal (LC), acoustic waves (TAG lens), and acoustic optics modulators (AOM)^8^ to achieve fast focal shifts (~1kHz). Ferroelectric LC and TAG lenses introduce a focal shift by varying the gradient of the refractive index of the liquid medium, however, the generated phase variation only approximates the defocus phase, leading to increased spherical aberration at large focal shifts^9–11^. AOM-based vari-focus techniques on the other hand use two AOMs with counterpropagating acoustic waves to cancel out the transverse scan but can only achieve focus shift in one dimension (acting as a cylindrical lens)^12,13^.

Adaptive optics-based vari-focus techniques overcome these limitations through accurate wavefront control using either a spatial light modulator (SLM) or a deformable mirror (DM), which can achieve a response rate of ~1 kHz and 20 kHz respectively. However, SLMs are polarization and wavelength-dependent and cannot model a continuous wavefront of the defocus phase due to its limited phase modulation depth. Large phase shifts are generated through multiple phase-wrapping of 2𝜋. With finite fly-back at the phase-wrapping borders, part of the incident light is not correctly modulated and results in decreased intensity at the focus^14^. DMs are not polarization and wavelength-dependent and can model a continuous defocus wavefront. However, the axial scan range of a DM is limited by the stroke length of the DM actuators. For example, for an objective with a numerical aperture (NA) of 0.8, the maximum axial scan range that DM based techniques can generate is ~40 µm^15^. Furthermore, using DM for focus control requires accurate alignment and complicated calibration of the DM to reduce the aberrations caused by imaging samples out of the nominal focal plane of the objective^9^.

Unlike the adaptive optics or DM-based approaches that require correcting the defocus plane-by-plane, pupil-matched remote focusing (pmRF), pioneered by Botcherby et al.^16,17^, instantaneously corrects defocus across 3D volumes for high-NA optics thereby conserving the microscope’s temporal bandwidth^16–26^. In addition, because pmRF allows precise mapping of the wavefront coupled into the back-pupil of the objective, where the angular magnification is unity, such techniques have been routinely used to carry out aberration-free high-quality axial focus control^16–26^. In pmRF techniques, a fast axial scan is achieved by the translation of a small mirror in front of the remote objective using a focus actuator^18,19,23^ or by a lateral scan of a galvo mirror in combination to a step or tilted mirror at the remote objective^27^. Because of the fast response time of the focus actuator or the galvo mirror, an axial scan rate of 1–5 kHz or 12 kHz can be achieved respectively. However, current pmRF techniques for focus control are primarily limited to the illumination path. This is because pmRF uses the concept of optical isolators^28^, where the polarization of the returning beam is rotated orthogonally to the incoming beam so that it can be separated from the incoming beam at the polarized beam splitter (PBS) (Supplementary Fig. 1a). *This configuration ensures minimum light loss through the pmRF module but requires the incoming beam to be polarized, which is why this method is primarily used in the illumination arm where illumination laser light is usually polarized in nature and its manipulation through the optical isolator can be easily done.* In the detection arm, however, the emitted fluorescence is unpolarized in nature. To the best of our knowledge, because, using purely linear optical elements, lossless conversion of unpolarized light into a single polarized state is not yet possible ^29,30^ (Supplementary Note 1), manipulating the fluorescent light using the optical isolators is unfeasible. As a result, microscopes that use pmRF to carry out axial scanning, incur 50% light loss due to one state of the polarized light being discarded after the PBS^16,21,24^ (Supplementary Fig. 1a).

A straightforward method to mitigate this problem is to have another copy of the pmRF module at the unused port of the PBS (Supplementary Fig. 1b) to collect the other half of the fluorescent light. However, this would require precise synchronization of two linear-focus-actuators (LFA), which is not only a difficult task at high speeds but also will be expensive since this method warrants two such LFAs. In this article, we present a novel optical design that overcomes these problems and presents a modular setup that can perform remote focusing on the detection arm of a fluorescent microscope without incurring polarization-induced losses. When attached to a light-sheet microscope, this technique allows optical refocusing without requiring the movement of the sample, or the detection objective (Fig. 1b and Supplementary Fig. 1c). As a result the microscope can acquire 3D volumetric data limited by camera speed. This technique is applicable to many other microscopy techniques that currently use an adjustable *Z*-stage to carry out volumetric imaging such as confocal, 2-photon, and light sheet variants.

## Results

### Concept and microscope layout

#### Optical axial-refocusing:

**Figure 1:**
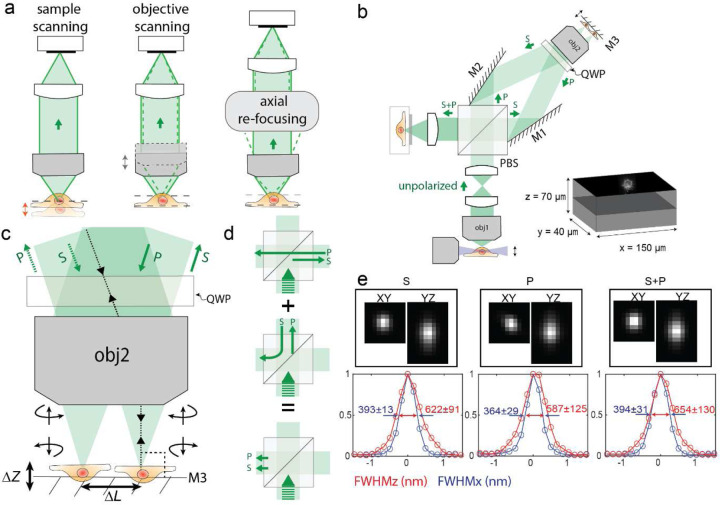
Schematic diagram of a remote focusing system implemented in light-sheet microscopy and its performance. a) Three different modalities to acquire volumetric imaging of the sample along the focus direction. Either the sample or objective lens can be moved for axial re-focusing. Alternatively, both the sample and objective lens can remain stationary by using a remote focusing system. b) Implementation of the remote-focusing system on the detection arm of the light sheet microscope. In this configuration, objective lenses 1 and 2 are pupil-matched through two lenses to form a perfect imaging system. Combined with mirror M3 and a polarizing beam splitter (PBS), the whole system works as a remote focusing system. The novel design of this remote-focusing system is implementation in the detection arm for unpolarized fluorescent light emitted from the sample. To do this, two tilted mirrors M1 and M2 are utilized to direct both S and P-polarized beams toward Objective lens 2 and then combine the reflected beams from mirror M3 to create an image by S and P-polarized beams onto the camera by focusing through the tube lens. The mirror M3 is attached to the linear focus actuator (LFA), moving back and forth to scan the sample in the *Z-*direction to acquire a 3D image. In the illumination arm, the generated light sheet by a cylindrical lens is translated by a galvanometric scan mirror (GSM) along the detection arm. To focus the detection path on the plane of the light sheet, the synchronization of GSM and LFA is carried out by sawtooth signals. Simultaneous dual-channel imaging of the cell is achieved in 40 µm×150 μm FOV over 70 μm in the *Z*-direction. c) The polarization state of the incoming beams changes after reflection from mirror M3 (S to P, and P to S). d) The reflected beams from mirror M3 have a different polarization state compared to the incoming beams; therefore, they exist from a different side of the PBS than the incoming beams. e) The point spread function (PSF) of 200 nm beads formed by S, P, and S+P polarized beams. The microscope performs at the diffraction limit, 394 nm resolution, for S, P, and S+P in the lateral directions (*X-Y*), while it maintains a resolution of 654 nm in the axial direction (*Z*).

Our refocusing unit is shown in Fig. 1b. Here, the water immersion detection objective (Obj1) is pupil matched to a second air objective (Obj2) through two intermediate lenses following the original design by Botceherby et al.^16,17^. However, unlike traditional refocusing geometry, we split the collected unpolarized fluorescence into S and P-polarized light using a polarizing beam splitter cube (PBS) in the infinity space of Obj2. The generated orthogonal paths are then projected onto Obj2 using two angled mirrors M1 and M2. Because of this angular launch in infinity space, Obj2 forms two distinct laterally shifted images at its nominal focal plane. A small mirror placed on an LFA reflects the light back through the path it came from where a quarter wave plate (QWP) converts the S-polarized light to P on its way back (and P-polarized light to S) after being reflected from the mirror (Fig. 1c). When the returning light (in each arm) reaches the PBS, it now acts as an optical valve where the S path (which was initially P) gets reflected while the P-polarized light (which was initially S) gets transmitted by the PBS. As a result, both S and P polarized light exits the PBS through the fourth and unused face of the PBS cube (Fig. 1d). This light after passing through a tube lens forms identical images, one with S and another with P, at the sCMOS camera. *A precise alignment using mirrors M1 and M2 overlays the two images, thereby resulting in a combined image by simply an incoherent addition without any interference artifacts.*

There are a few important design considerations that need to be considered for our de-scanning setup. Firstly, it is essential that mirror M3 consistently moves in parallel with the focal plane of Obj2 during the LFA’s oscillatory motion. This prevents any unwanted focal shifts between the S and P paths, ensuring that the resulting image from both S and P polarizations remain focused on the camera at the same time. This arrangement ensures that both beams return through their incoming paths, resulting in easier alignment for overlaying the final images formed by the S and P-polarized beams.

Secondly, it is advantageous that θ (angle between S and P polarized beam hitting the Obj2) (Supplementary Fig. 2) be as small as possible because this directly controls the distance between the two focal points at M3 (depicted by ∆𝐿 in Fig. 1c). A smaller ∆𝐿 ensures: (1) a smaller mirror could be utilized to carry out the remote-focusing, reducing the inertial load on the LFA, and enhancing its efficiency; (2) The alignment becomes less sensitive to tip-tilt misalignment of M3; and (3) This guarantees that both images fit within Obj2’s field of view (FOV).

Thirdly, there exists an inverse relationship between the angle θ and the distance between Obj2 and the PBS (inset of Supplementary Fig.2). Therefore, this gives us an option: *either adhere to the 4f system or minimize θ*. We found that for our matching objectives Obj1 and Obj2 the 4f system (with matching lenses L1 and L2) resulted in a θ of 20⁰ (inset of Supplementary Fig.2). However, operating in this range poses a risk as it is challenging to ensure that both reflected beams are entirely captured by Obj2. Hence, there is a balance between adhering to the 4f system and minimizing the angle θ. We found that with our current design, we can still achieve diffraction limited resolution (Fig. 1e).

Finally, because we generated two identical images on the camera using S and P-polarized light, it was crucial to overlay these images with precision higher than the diffraction-limited resolution to produce the final image. To do this, we developed a cross-correlation-based algorithm that quantifies the shift between overlayed S and P images in real-time with sub-pixel accuracy, allowing interactive adjustment of the mirrors M1 and M2 during system alignment.

#### Implementation in a light-sheet system:

In order to test the performance of our design we implemented this setup into the detection arm of a light-sheet microscope with orthogonal illumination and detection objectives. The system layout is shown in (Fig.1b and Supplementary Fig.2). The sample is illuminated by a sheet of light generated with a cylindrical lens in the illumination arm, and the emitted fluorescence from the sample is collected by the detection objective lens, which is set orthogonal to the illumination objective lens to capture 2D information from the sample. A galvanometric scan mirror (GSM) in the illumination arm translates the light-sheet in the *Z*-direction. Because the position of the LFA in the detection arm determines the focal plane of the detection objective lens, we synchronized the GSM and LFA with the sawtooth signal to ensure that the detection path is always focused on the plane of the light-sheet (Supplementary Fig.3). This allowed us to carry out volumetric imaging by acquiring a sequence of images from different focal planes. The LFA moves back and forth rapidly, synchronized with the movement of the GSM enabling us to quickly collect 3D image stacks.

The optical correction of defocus in our high-NA microscope allowed fast de-scanning of a 3D volume over an axial range of ~70 µm at speeds limited primarily by the camera framerate(~in our case 799 camera frames/s at 2304 ×256 pixels using Hamamatsu Orca-fusion BT). We employed a dual-color imaging strategy by partitioning the FOV, enabling simultaneous capture of two distinct fluorescent labels within each slice without sacrificing imaging speed. To do this we used a pair of dichroic mirrors to separate the emitted wavelengths from the two labels into side-by-side dual-color images (Supplementary Fig.2). Once acquired, these separate image sets are then precisely registered and merged to generate 4D (*X, Y, Z,* and *λ*) stacks. By sequentially capturing 4D stacks, we generated 5D (X, Y, Z, λ, and time) datasets that allowed us to track the dynamic behavior of biological processes. It is important to note that our setup is wavelength-independent, an attribute not feasible with technologies like diffractive tunable lenses or spatial light modulators.

### Characterization of the optical system

**Figure 2:**
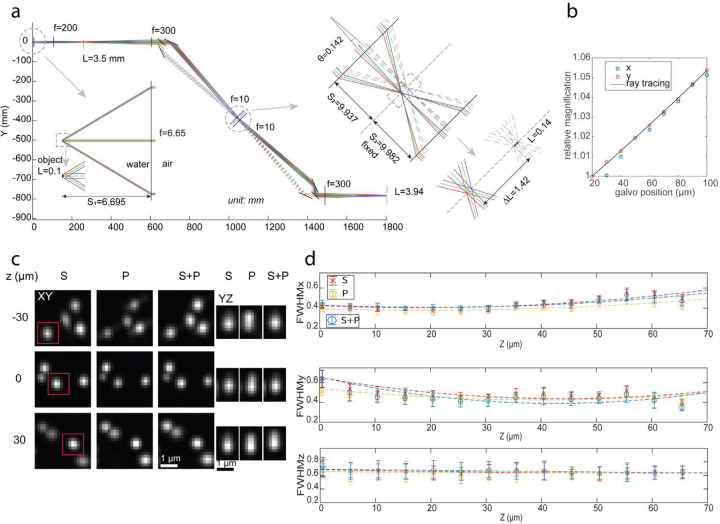
Ray tracing of the setup and resolution assessment. a) Ray tracing of the detection path. L: image size, f: effective focal length, S: image or object position relative to the lens, unit: mm. b) Calibration of lateral magnification at various object positions, a target illuminated by a white light LED is imaged for magnification measurement. c) Maximum intensity projections of data acquired on 200 nm beads from 10 slices spaced 500 nm in the *Z*-direction. The images show orthogonal views of the MIPs across scan range for S,P and S+P. The elongated PSF in the *Z* direction exhibits less resolution in the axial direction controlled by the light sheet waist. d) The FWHM of the 200 nm beads in the lateral and axial directions over the scan range. The minimum lateral resolution, 394 nm, occurs at the center of the scan range and increases by moving away from the center. These plots show a constant axial resolution of 650 nm over the axial scan range. The microscope functions in the scan range of 70 µm.

To understand the image formation of the proposed setup, we simulated the ray tracing of the detection path (Fig. 2a). The ray tracing assumes all rays satisfy paraxial approximation and all lenses are simple lenses. The detection objective is a water immersion objective, we calculated its effective focal length as $f_{\text{obj}}=f_{\text{Tube}}n/M_{\text{obj}}$, where $f_{\text{Tube}}$ is the focal length of the designed tube lens, $M_{\text{obj}}$ is the magnification of the objective, and *n* is the refractive index of water. Here we have $f_{\text{obj}}$ equal to 6.65 mm. The pmRF module (from the beam splitter to LFA) is modeled two times to simulate the forward and backward transmission through the module. The LFA is omitted from the simulation, instead, we change the distance between the two copies of the pmRF objectives so that the distance (*S*_3_) of the image plane to the second pmRF objective remains as a constant. We simulated with an object of 100 µm, the image size after the pmRF objective is ~140 µm, indicating a lateral magnification of 1.4, which is close to the requirement of perfect imaging with $M_{\text{lateral}}=n_{\text{water}}/n_{\text{air}}=1.33$. The small deviation is limited by the geometry of the pmRF module: the separation (ΔL) of the S and P-images formed by the pmRF objective is approximate to $\text{Δ}L=f_{\text{RFobj}}\theta$, where $f_{\text{RFobj}}$ = 10 mm is the effective focal length of the RF objective and θ is the angle between the S and P-polarized rays meeting at the RF objective. The larger the ΔL, the larger the aberration introduced by the pmRF objective. To reduce ΔL, the pmRF objective is located ~500 mm from the PBS, therefore, the pmRF module is no longer an exact 4f system, the magnification, $M_{\text{lateral}}$, varies with the axial position of the object. Furthermore, the beam path from the detection objective to the tube lens is also not a 4f system, where the tube lens is ~100 mm away from the detection objective. The combination of the two non-4f systems can partially reduce the axial dependence of the magnification. Fig. 2b shows the change of the lateral magnification with respect to the galvo position (the axial position of the light sheet) from both ray tracing and the experimental data. There is a ~5% magnification change over an axial range of 80 µm. This magnification change can be further reduced by optimizing the axial position of the tube lens.

To quantify the performance of the proposed scheme, we used full width at half max (FWHM) measurements of 3D point spread function (PSF) to validate that the incoherent addition of S and P images was not compromising the resolution. To do this, we measured the PSF of each polarization component individually and compared it with the PSF of the unified S+P image. As illustrated in Fig.1e, both the S and P-polarized images rendered onto the camera exhibit identical FHWM, resulting in an equivalent resolution for the combined S+P image. Further quantification involving 10 randomly chosen beads, reveals that the microscope achieved diffraction-limited resolutions: 394±31 nm laterally (*X-Y*) and 654±130 nm axially (*Z*). These measurements were performed in proximity to the nominal focal plane (MIP of 10 slices, each separated 500 nm).

To evaluate the performance of the de-scanning system, we imaged 3D volumes of 200 nm beads embedded in a 2 % agarose cube across the scan range and accessed the quality of the generated PSFs. Fig. 2c shows the maximum intensity projection (MIP) of beads (from 10 axial slices, each slice spaced 500 nm) separated by 30 µm for S, P, and S+P across the scan range, after 10 iterations of Richardson-Lucy (RL) deconvolution. We found that our remote focusing setup demonstrated close to diffraction-limited performance over a scan range of ~70 um. As evident from the ‘S’ and ‘P’ images the quality of the beads visually appears similar across the entire scan range thereby resulting in an identical ‘S+P’ image. In the axial direction (the *YZ* view) the PSFs are limited by the Gaussian light sheet’s waist (beads from red boxes in *XY* view), which was determined by the tradeoff that exists between the FOV and *Z* resolution. We found that in order to image an entire cell, we needed a lightsheet that would generate a FOV of ~8 µm (Supplementary Fig. 4). As a result, we reduced the NA of the illumination objective and chose a light sheet whose waist was at FWHMz of ~ 650 nm after deconvolution (850 nm before deconvolution).

Figure 2d displays the measured FWHMs from 200 nm beads after RL deconvolution for S, P, and S+P polarize images in the lateral (*XY*) and axial (*Z*) directions across the entire scan range. The figure shows a minimum lateral FWHM of 394 nm at the center of the scan range which slowly increases as the beads move away from the nominal focal plane. This can be attributed to residue index mismatch aberrations that were not corrected by the remote focusing system^21^. Additionally, we found that the S polarization path suffered more in lateral resolution compared to the P polarization path and the trend is different along *X* and *Y* directions. This asymmetric FWHMs (*X-Y*) across scan range (*Z*) and the discrepancy between S and P paths is likely due to field-dependent aberrations from Obj2, where the S and P images were formed at different field points of Obj2 (Fig. 1b). Furthermore, our microscope shows a constant axial FWHM of ~ 650 nm over the entire scan range as the axial resolution is mainly determined by the light-sheet waist.

### Fast 3D live cell imaging

As a first demonstration of the 3D cellular imaging capabilities, we monitored the 3D motion of secretory granules in living mast cells. Mast cells possess distinct secretory granules that contain the mediators of the allergic response and are released upon mast cell activation by allergen^31^. These granules are distributed across the cytosol and have been shown to undergo both Brownian diffusion and directed motion^31^. Upon activation of the membrane receptor, FcεRI, via crosslinking by multivalent antigen^32,33^, the granules undergo increased directed motion that moves them to the plasma membrane where they will fuse and release mediators that regulate allergic responses^31,34^.

**Figure 3:**
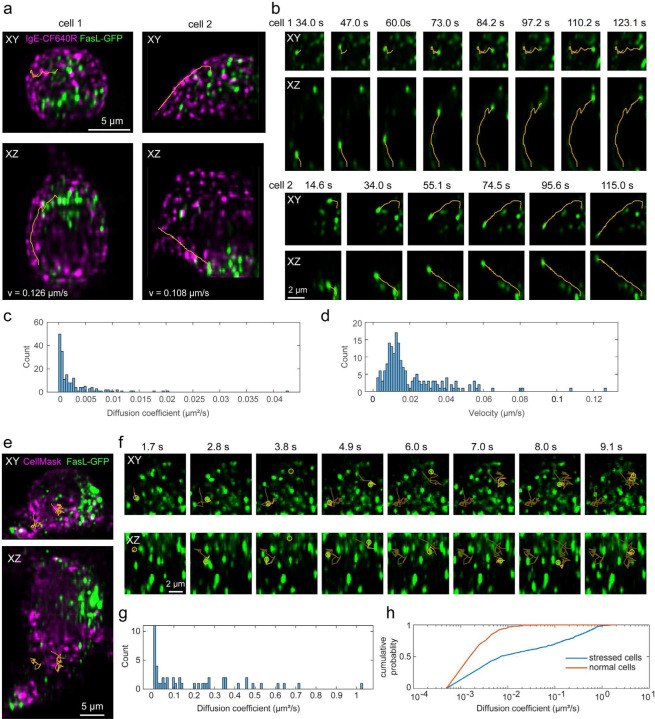
Dual-Color volumetric imaging of live RBL cells. (a-d) Dual-color volumetric imaging of granule motions in a live RBL-2H3 GFP-FasL cell, where the cell membrane is labeled with IgE-CF640R and granules contain GFP-FasL, at an imaging speed of ~ 0.6 volumes (80×15×40 µm^3^ in *XYZ*) per second for 80 volumes, for a total imaging time of ~2 minutes. (a) Maximum intensity projection views of the cell images at one-time point and overlay with representative trajectories of granule movement (orange lines). (b) Time series of the trajectories in a. (c, d) Histograms of estimated diffusion coefficients and velocities of all trajectories found in cell 1 and cell 2. (e-g) Dual-color volumetric imaging of live RBL-2H3 GFP-FasL cell, where the cell membrane is labeled with CellMask DeepRed and the granules contain GFP-FasL using an imaging speed of ~8.3 volumes/s for 80 volumes for a total time of 10 s. (e) Maximum intensity projection views of the cell images at one-time point and overlay with representative trajectories of granule movement (orange lines). (f) Time series of the trajectories in e. (g) Histograms of estimated diffusion coefficients of all trajectories in the cell. (h) Cumulative probability of the estimated diffusion coefficients under normal (a-d) and stressed (e-g) imaging conditions. 400–500 trajectories with a diffusion coefficient > 0.001 µm^2^/s from four cells under each condition are selected.

We applied the developed system for dual-color, volumetric imaging of live cells and tracked the 3D motion of green fluorescent protein-labeled Fas ligand (GFP-FasL) loaded secretory granules in the cytosol of RBL-2H3 mast cells ^31^. IgE-bound FcεRI was simultaneously imaged by addition of anti-DNP IgE-CF640R. With addition of the antigen-mimic, DNP-conjugated to BSA (DNP-BSA), FcεRI aggregates and undergoes endocytosis as seen in Figure 3a. During data acquisition, the light sheet is parallel to the *XY* plane and scans along the *Z* direction. Within the light-sheet region, the *XY* and *XZ* maximum intensity projections (Fig.3a) of the cell image show GFP-FasL granules in three dimensions. The cells were imaged at ~ 0.6 volumes (80×15×40 µm^3^ in *XYZ*) per second for 80 volumes, for a total imaging time of ~2 minutes (Fig.3a-d). To quantify the granule dynamics, isolated granules were identified and tracked in 3D using the U-track3D software^35^. We calculated the mean square displacement (MSD) of each trajectory over time and extracted the diffusion coefficient, *D*, and velocity, *v*, by fitting the MSD curve with $MSD\left(t\right)=6Dt^{2}+v^{2}t^{2}+o$, where o is an offset related to localization and tracking uncertainties^36,37^ (Fig.3c,d). We found that most granules undergo Brownian Diffusion and a few exhibited directed motion, consistent with granules being transported along the microtubules (Fig.3a,b)^31^. The measured transport velocities of the two trajectories indicated in Fig.3a,b are ~0.1 µm/s, consistent with previous work that performed tracking in 2D^31^.

To test the limits of the new system in terms of speed, we set out to image Brownian motion on the microscopic level. For this, we stressed the cells by incubating them in Hank’s balanced salt solution (HBSS) (Method) at room temperature for over 1 hour, which induced cell blebbing. This also caused more rapid diffusion of the granules that we were able to capture using an imaging speed of ~8.3 volumes/s for 80 volumes for a total time of 10 s. With this imaging speed, we retained good signal-to-noise and the ability to track the 3D motion of individual granules (Fig 3e-g). Under these non-physiological conditions, average granule diffusion was increased by ~41 times (Fig.3h). Two tracks shown in Fig. 3e have diffusion coefficients of 0.41 µm^2^/s and 0.64 µm^2^/s.

## Discussion

In this work, we developed an axial scanning module in the detection path of a light-sheet microscope utilizing the pmRF technique proposed by Botcherby et al.^16,17^. While inheriting all the benefits from the pmRF technique, such as fast scanning and all-optical aberration compensation (no wavefront control element), our design overcomes a critical limitation of the original pmRF technique, as in, the loss of 50% of the emitted fluorescence in the detection path^21,24,38^. Here we engineered a new optical design, where we split the emitted fluorescence into S and P polarized light to carryout remote focusing and then seamlessly combine them to achieve minimum light loss. We demonstrated our implementation of the developed scanning module through a light-sheet microscope with two orthogonally arranged objectives. We can perform simultaneous two-color imaging at 8.3 volumes (80×15×40 µm^3^ in *XYZ*) per second with a lateral resolution of 394 nm and an axial resolution of 650 nm (after deconvolution). As our method is fully optical, the imaging speed scales with advancements in LFA technology and camera acquisition speed.

The S and P polarized beams are directed at an oblique angle into the remote objective (Fig. 1b). This angled approach creates two separate images at the mirror attached to the LFA (M3). However, there are limitations to this angular arrangement. The two images formed away from the optical axis are prone to aberrations. To reduce the image separation, the remote objective must be positioned further from the PBS to reduce the incident angles of S and P-polarized lights. However, this increased distance breaks the 4f configuration between the two objectives (detection and remote objectives) that is critical to achieving aberration-free imaging. Future studies will investigate into more compact designs that will better satisfy the 4f condition and will reduce the separation between the two foci at M3.

Of note, our approach offers several advantages over the existing axial refocusing methods. First, it provides an extended, aberration-free scan range for high numerical aperture (NA) optics. This is a significant benefit when compared to techniques based on deformable mirrors (DMs), where our method approximately doubles the axial scan range of DMs^15^. Second, it is wavelength independent, which makes it suited for simultaneous multicolor imaging when compared to SLMs and tunable lenses. Additionally, unlike SLMs which depend on polarization, our arrangement is not dependent on the polarization of the fluorescence. Furthermore, unlike SLMs, which are typically slow (especially the nematic liquid crystal ones), and even their faster counterparts (ferroelectrics) tend to be less effective, our method allows for imaging speed that are only limited by the sCMOS’s framerate.

Although recent advancements in single-objective oblique plane microscopy (OPM) have achieved speeds comparable to our method, our technique presents several notable advantages. In OPM, the de-scanning of the returning fluorescent light leads to skewed images. Before these images can be viewed, they require intensive de-skewing processes^2,39–43^.On the other hand, our approach captures 3D volumes in a conventional orthogonal setup. This is achieved by recording high-speed images while sweeping the light-sheet through the sample. Each frame captured by the camera represents an optical cross-section of the specimen. As a result, the 3D image stacks generated using our method are immediately available for viewing. They may benefit from an optional deconvolution, but there’s no delay caused by necessary post-processing. Furthermore, the OPM setup necessitates a third objective, which in the latest setups require expensive objectives like ‘Snouty’ or ‘King Snout’^2,39–42^. Our setup on the other hand does not have this requirement and our secondary objective performs the role of a tertiary objective. Moreover, while not demonstrated explicitly here, our method can be employed to achieve isotropic resolution, a feat the OPM cannot achieve.

Compared with the original Botcherby’s remote focusing setup, our pmRF module folds the beam path between the detection and the remote objectives. This configuration complicated the optical alignment. A potential solution is to arrange both objectives inline in a 4f configuration. Furthermore, we note that although an all-optical design has its merit of simplicity and robustness, using an objective lens in the pmRF module introduces ~30–40% light loss (Supplementary Fig.5) compared with the axial scanning techniques based on DMs, future development of objective with high-transmission efficiency is desirable.

Finally, it is our firm belief that owing to its generalized design, we envision our method has the potential to transform many popular microscope modalities like confocal, 2-photon, and the rapidly emerging field of light sheet microscopy, by reinventing how they perform scanning in the axial dimension.

## Methods

### Optical setup

The illumination arm consists of two laser sources (Coherent Sapphire 488 nm and Obis LX 637 nm) which were combined with a dichroic beam splitter (LM01–503-25, Semrock). To clean up the beams, the beams were focused through a 50-µm pinhole (P50D, Thorlabs) by a 45-mm achromatic doublet (AC254–045-A, Thorlabs) and then recollimated using a 150-mm achromatic doublet (AC254–150-A-ML, Thorlabs). The original beams were expanded by 9 folds with a 3× Galilean beam expander (GBE03-A) before being focused with a cylindrical lens (ACY254–50-A, Thorlabs), onto a resonant mirror galvanometer (CRS 4 kHz, Cambridge Technology), driven by a 12-volt power supply (A12MT400, Acopian), to wobble the light sheet. One-dimensional focus was then recollimated with a 100-mm achromatic doublet (AC254–100-A-ML, Thorlabs) and hit the galvanometric scan mirror (GSM) (GVS111, Thorlabs), driven by a 15-volt power supply (GPS011, Thorlabs), for rapid shifting of the light sheet along the detection arm. This galvanometric mirror was conjugated to the back pupil of the objective lens (Nikon 40x/0.8 NA) through 100-mm and 200-mm achromatic doublet (AC508–100-A-ML and AC508–200-A-ML, Thorlabs).

In the detection arm, the same objective lens (Nikon 40x/0.8 NA) in an orthogonal setup was used and pupil-matched to the scanning objective lens (Nikon Plan Apo 20x/0.75 NA) through a 200-mm tube lens (TTL200-A, Thorlabs) and a 300-mm achromatic doublet (AC508–300-A-ML, Thorlabs). A 50:50 polarizing beam splitter (PBS) (10FC16PB.3, Newport), splits the beam in S and P polarized light. Using mirrors M1 and M2 these light paths where then launched at an angle towards the Obj2 (Supplementary Fig.2). It is extremely critical to minimize the angle of the launch. Both experiment and simulation predicted that we used 8 degrees as the launch angle (called θ) (Fig.2a, inset of Supplementary Fig.2). The S and P polarized light passed through a quarter waveplate (AQWP10M, Thorlabs) and were focused onto a mirror positioned at the focus of the scanning objective lens. The mirror (PF03–03-P01 - Ø7.0 mm Protected Silver Mirror, Thorlabs) was attached to a voice coil with a travel of 10 mm, positional repeatability of fewer than 50 nanometers, and a response time of fewer than 3 milliseconds (LFA-2010, Equipment Solutions). Then the reflected light was recaptured by the same scanning objective lens and quarter-wave plate to rotate the beam’s polarization state. Afterward, the light was directed toward an sCMOS camera (Hamamatsu Orca-fusion BT) by reflecting from the same cube polarizing beam splitter and a 300-mm achromatic doublet (AC508–300-A-ML, Thorlabs). For emission filters, we used two long-pass filters (FF01–525/30–25, and BLP01–647R-25, Semrock), for blue, and far-red, respectively. To image dual channels simultaneously, the field of view (FOV) was separated into half using dichroic mirrors (DMLP605R, Thorlabs) between the 300-mm achromatic doublet and the camera. We immersed the specimen and the illumination and detection objectives in a chamber designed using Adobe Inventor and machined through Protolabs (R). The LFA and GSM, in the detection and illumination arms respectively, were synchronized together to always keep the translated light sheet in the focus of the detection objective lens to acquire a 3D stack of the specimen.

### Overlaying S and P images with sub-pixel accuracy

Two identical images -- one corresponding to S and another to P polarized light -- are formed at the camera and added incoherently to generate the final image. We used a custom-written MATLAB script to monitor the offset between the two images in near real-time while adjusting the positions of M1 and M2 (Supplementary Fig.6).

The offset between the two images using a cross-correlation-based algorithm as was used in Wester, M.J. et al^44^, which achieves sub-pixel accuracy by fitting second-order polynomials through the peak of the scaled cross-correlation between the S and P polarized images.

Initially, an image is acquired as a reference by obstructing one optical path (either S or P). Subsequently, the alternative optical path is used to collect new images. The shift between each new image and the reference image is then measured using the method described in Wester, M.J. et al^44^ and is available as the MIC_Reg3DTrans.findStackOffset method in the matlab-instrument-control toolbox^45^. And while new images are collecting, mirrors M1 and M2 are adjusted to minimize the shift.

### Microscope control

A Dell Precision 7920 computer with two processors Intel(R) Xenon(R) Silver 4210R CPU having a processing speed of 2.40 GHz and 2.39 GHz and was integrated with 128 GB RAM was used to acquire the microscope’s data. An NVIDIA Quadro RTX 4000 Graphics processing unit (GPU) with dedicated memory of 8 GB and shared memory of 63.8 GB (GPU memory of 71.8 GB) was also integrated into the system. 64-bit operating system ×64-based processor facilities the system to operate. LabView 2020 64-bit allowed us to work with the required software, including the LabView Run-Time Engine, Vision Run-Time Module, Vision Development Module, and other required drivers like NI-RIO drivers (National Instruments). DCAM-API software was used for the Active Silicon Firebird frame-grabber to actively interfere with the scientific complementary metal-oxide semiconductor (sCMOS) camera (ORCA-Fusion BT Digital CMOS camera, model: C15440–20UP) manufactured by Hamamatsu, Japan. It generated deterministic transistor logic (TTL) trigger sequences through 150 Watts shutter instrument (100–240 V~50/60 Hz; model: MP-285A) with a field programmable gate array (FPGA) (PCIe 7852R, National Instruments). The generated triggers controlled the resonant mirror galvanometers, placement of the stage, voice coils, blanking and modulation of laser, firing camera, and other external triggers. K-Hyper Terminal software facilitated engaging LFA with the system hardware. Some key features along with some routines under the agreement of material transfer were licensed by the Howard Hughes Medical Institute’s Janelia Farms Research Campus.

### Sample preparation

Bead sample:200 nm beads embedded in 2% agarose gel was used for microscope resolution assessment. To make 2% agarose gel, 2 g of agarose powder (A9045–25G, SIGMA life science) was mixed with 100 mL water and swirled thoroughly before putting into the microwave oven to heat. Once the solution boiled and got completely clear and the agarose was dissolved, we should remove the solution from the oven and let it cool down. Then, 200 nm beads (YG, Polysciences) were mixed with water with a ratio of 1/100 to form a solution of the 200 nm beads. It was sonicated before mixing with the molten 2% agarose gel with a volumetric ratio of 1/10. Then this molten combination was poured into the cubic mold where the sample holder was placed there and sat there for a few minutes to dry and form a 1cm^3^ cubic sample (200 nm beads embedded into 2% agarose gel) attached to the sample holder.

Cell samples: RBL-2H3 GFP-FasL cells were cultured in Gibco Minimum Essential Media (MEM) media supplemented with 10% heat-inactivated Fetal Bovine Serum (FBS), 1% Penicillin/Streptomycin, and 1% L-glutamine^31^. The cells were primed with 1 µg/ml anti-DNP-IgE^46^ (Fig. 3e-g) or anti-DNP-IgE-CF640R (Fig. 3a-d) and seeded at a density of 100,000 cells per well in 12 well dish over 5 mm glass coverslips and incubated with 5% CO2 at 37°C overnight. IgE-CF640R was prepared using CF640R NHS-ester (Biotium #92108). For fast imaging experiments, the cellular membranes of anti-DNP-IgE primed cells were labeled with CellMask^™^ Deep Red Plasma Membrane Stain (Thermo Fisher Scientific #C10046, 5mg/ml, 1000X) according to manufacturer’s instruction for 10 minutes in modified Hank’s balanced salt solution (HBSS) (additional 10 mM Hepes, 0.05% w/v BSA, 5.45 mM glucose, 0.88 mM MgSO4, 1.79 mM CaCl2, 16.67 mM NaHCO3) and rinsed with HBSS. Cells were stimulated with 1 µg/ml DNP-BSA in the sample chamber. Data was acquired in 2 min captures for up to 15 minutes post antigen treatment.

### Sample mounting

Cells samples on 5 mm coverslips were loaded onto the holder as depicted in Supplementary Fig.7. In the sample holder, two metal wires were designed to clamp the coverslip tightly. This sample holder was attached to the *XYZ* Translation Stage with Standard Micrometers using a rotation mount. As a result, the coverslip had four degrees of freedom, including the translation on the *X-Y-Z* axis to locate the cells while imaging and the rotation around the X-axis to face the coverslip with the desired angle relative to the illumination and detection objectives. Here, the coverslips were faced 8 degrees relative to the optic axis of the detection objective (Supplementary Fig.7). In order to minimize the buffer volume for live cell imaging, the 6 ml chamber was designed to immerse the sample, illumination, and detection of objective lenses into it (Supplementary Fig.7).

### Image processing pipeline

Data were analyzed with the custom script written in Matlab. The procedure for quantifying the microscope’s resolution from fluorescence bead data is as follows: 1) A 3D-PSF model was generated from the raw data. 2) The light-sheet region was cropped from each slice of the raw data. The width of the light-sheet region was defined by the distance to the waist of the light-sheet where the axial resolution increases by 2 times. We sat the light-sheet width to be ~6 mm. Note that the light-sheet region translated along its width direction (the *Y*-axis) while it was being scanned in the axial direction (*Z*-axis relative to the detection objective) (Supplementary Fig.8). Therefore, the light-sheet region to be cropped was also shifted in *Y* accordingly (Supplementary Video 1). 3) the cropped region was deconvolved with the 3D-PSF model using Richard-Lucy deconvolution from ImageJ. 4) the deconvolved data stack was divided into segments with an axial dimension of 5 µm. For each segment, candidate beads were selected and their FWHMs along each dimension were estimated from Gaussian fitting of their intensity profiles along that dimension. 5) the measured FWHMs were used to quantify the resolution of the microscope as shown in Fig. 2d.

Dual-color live-cell data was processed as follows: 1) cell signal from each color channel was cropped with a user-selected region. 2) for each color channel, the *XYZ* drifts of the data stack at each time point relative to the reference data stack were estimated, where the maximum-intensity projection (MIP) along each dimension of the two data stacks was generated and the 2D shift between each pair of the MIP images was calculated through cross-correlation. 3) an average of the *XYZ* shifts from both channels was used to correct the drift between time points. 4) the *XYZ* shift between the two-color channels was calculated by first averaging over the time dimension for each color channel, then estimating the shift from the MIP images as in step 3. Then register the two channels by applying the estimated shift. 5) after drift correction and channel registration, the resulting image stacks were deconvolved with the 3D-PSF model generated from the bead data using Richard-Lucy deconvolution from Matlab. 6) to reduce noise and correct photo-bleaching, the deconvolved images were subtracted by a background value with negative pixel values set to zero and divided by a normalization factor equal to the 99.95 quantiles of all pixel values in the corresponding time points and color channel.

### Quantification of Light-sheet dimension

To quantify the light-sheet dimension, bead data in agarose gel were collected at different slit widths. At each slit width, we estimated the FWHMs in *XYZ* for all selected beads as described above, however, here we used the full FOV of the color channel for bead imaging. As the position of the light-sheet waist shifted in y with respect to the axial dimension, we corrected the y coordinates of the selected bead by $y_{cor}^{\prime }=y_{cor}-az_{cor}$, where *a* is the y shift by moving one pixel in *Z* (Supplementary Fig.8). Then we fitted the $FWHM_{z}$ verse $y_{cor}^{\prime }$ for all selected beads with a polynomial function (Supplementary Fig.4). The length of the light-sheet was found when the $FWHM_{z}$ was twice the minimum from the polynomial fit.

### Magnification calibration

One brightfield image of the calibration target was captured at each of the galvo positions from −40 to 40 μm with a step size of 10 µm. The target image consisted of parallel line segments, we cropped a region of 700×700 pixels from each image (Supplementary Fig.9a-b). We then calculated the affine transformation (from the Dipimage toolbox) of each image with respect to a reference image. The zoom factors from affine transformation were used to quantify the relative magnification between each image to the reference image. The absolute magnification of one image was calculated as follows: crop a narrow section of multiple parallel lines, obtain the intensity profile by averaging over the line dimension, smooth the intensity profile by applying a running average with a window size of 30 pixels, find all peaks from the smoothed intensity profile (Supplementary Fig.9c), calculate the average distance (in pixels, denoted as ∆*d*) between consecutive peaks, as the distance between consecutive parallel lines is 10 mm, then the pixel size at the sample plane can be estimated from 10/∆*d* mm, therefore the magnification can be calculated from the pixel size of the camera divided by pixel size at the sample plane.

### Ray tracing

Ray tracing was based on geometric optics with paraxial approximation. The ray propagation was calculated using the ABCD matrices. Two matrices were used, the translation matrix,

$$M_{d}=\left[\begin{array}{cc} 1 & 0\\ d/n & 1 \end{array}\right],$$

and the matrix of a thin lens,

$$M_{f}=\left[\begin{array}{cc} 1 & -n/f\\ 0 & 1 \end{array}\right],$$

where d is the translation distance, f is the focal length of the thin lens and *n* is the refractive index of the propagation medium. For our system, n is 1.33 before the detection objective (including the objective) and n equals 1 for the rest of the ray tracing. The starting point of each ray was represented by a vector of ${\left[n\alpha ,y\right]}^{T}$, where α and y are the angle and the y position of the ray with respect to the optical axis. The propagation of the ray is then calculated from

$$\left[\begin{array}{c} n\alpha \prime \\ y\prime \end{array}\right]=M\left[\begin{array}{c} n\alpha \\ y \end{array}\right].$$

For a defined FOV, we selected three field points, two mark the edge of the FOV and one at the optical axis. For each field point, we generated three rays at different angles that will intersect three points at the pupil plane, where two points mark the edge of the pupil and one at the center of the pupil. Rays from the same field point were colored the same. The optical axis after the polarizing beam splitter was rotated by 45 degree to be along the splitting plane of the PBS. The ray tracing after the PBS was done by first transforming the ray coordinates to the ones defined by the optical axis and propagating the ray with the ABCD matrix, then transforming back to the global coordinates. Except for the distance between the tube lens and the detection objective (denoted as *d*_1_ ), the rest distances between consecutive optical elements were measured with a ruler. The angle between the chief rays of the S and P-polarization (𝜃𝜃 in Fig.2a) was set when the input and output beam diameters at the remote focusing objective were minimum. The distance *d*_1_ was set when the relative magnifications from ray tracing match with the measured ones (Fig. 2b). The central position of the scan range, the distance of the objective to the detection objective (denoted as *S*_1_), was set when the absolute magnification from ray tracing matches with measured one. Here *S*_1_ = 6.695 mm, which was 45 µm away from the designed focal plane of the detection objective.

## Supplementary Material

Supplement 1
