# Supplementary material for: Axial de-scanning using remote focusing in the detection arm of light-sheet microscopy
Source: Res Sq. 2023 Oct 3:rs.3.rs-3338831. Preprint. [Version 1] doi: 10.21203/rs.3.rs-3338831/v1 (PMC10602066; doi:10.21203/rs.3.rs-3338831/v1)
Supplement: Supplement 1 [file NIHPPRS3338831V1-supplement-1.pdf]

Supplementary Files

This is a list of supplementary files associated with this preprint. Click to download.

- SupplementalVideo1.avi
- SupplementaryFileSept1.pdf
